# Allele-specific recognition by LILRB3 and LILRA6 of a cytokeratin 8 - associated ligand on necrotic glandular epithelial cells

**DOI:** 10.18632/oncotarget.6905

**Published:** 2016-01-13

**Authors:** Des C. Jones, Colin R.A. Hewitt, María R. López-Álvarez, Martin Jahnke, Alasdair I. Russell, Valeria Radjabova, Alice R.Z. Trowsdale, John Trowsdale

**Affiliations:** ^1^ Department of Pathology, University of Cambridge, Cambridge CB2 1QP, UK; ^2^ Department of Genetics, University of Leicester, Leicester LE1 7RH, UK; ^3^ Cancer Research UK Cambridge Institute, University of Cambridge, Li Ka Shing Centre, Cambridge CB2 0RE, UK

**Keywords:** cytokeratin, LILR, breast cancer, immune escape, DAMP

## Abstract

The LILRs are a family of receptors that regulate the activities of myelomonocytic cells. We found that specific allelic variants of two related members of the LILR family, LILRB3 and LILRA6, interact with a ligand exposed on necrotic glandular epithelial cells. The extracellular domains of LILRB3 and LILRA6 are very similar and their genes are highly polymorphic. A commonly occurring allele, LILRB3*12, displayed particularly strong binding of these necrotic cells and further screening of the products of LILRB3 alleles identified motifs that correlated with binding. Immunoprecipitation of the ligand from epithelial cell lysates using recombinant LILRB3*12, identified cytokeratins 8, 18 and 19. Purified proteins obtained from epithelial cell lysates, using anti-cytokeratin 8 antibodies, were able to activate LILRB3*12 reporter cells. Knock-down of cytokeratin 8 in epithelial cells abrogated expression of the LILRB3 ligand, while staining with recombinant LILRB3*12 showed co-localisation with cytokeratin 8 and 18 in permeabilised breast cancer cells. Necrosis is a common feature of tumours. The finding of a necrosis-associated ligand for these two receptors raises the possibility of a novel interaction that alters immune responses within the tumour microenvironment. Since LILRB3 and LILRA6 genes are highly polymorphic the interaction may influence an individual's immune response to tumours.

## INTRODUCTION

Necrotic cell death commonly occurs in tumour cells where it may be a consequence of impaired apoptosis (programmed cell death). Necrotic cells release large amounts of intracellular molecules exhibiting Damage-Associated Molecular Patterns (DAMPs) that alter the immune response upon recognition by professional antigen presenting cells (APC) such as macrophages, monocytes and dendritic cells (DC).

The leukocyte Ig-like receptors (LILR), encoded in the leukocyte receptor complex, are a family of innate immune receptors that regulate the behaviour of myelomonocytic cells [1, 2]. The LILR gene complex encodes multiple receptors with either inhibitory functions, determined by ITIM motifs in their cytoplasmic domains, or activating functions, due to their association with ITAM-bearing adaptor molecules. The competing activities of activating and inhibitory members of the LILR family are thought to finely balance the functions of innate immune cells and dictate their response to infected, stressed, transformed and normal cells. Members of the LILR family are involved in the control of human DC maturation, cytokine production and the expression of co-stimulatory molecules that direct adaptive immunity [3-7].

LILR are divided into two groups based on their ability to bind MHC Class I molecules [8]. Much is already known about the ligands of group 1 LILR, which are MHC class I [9], but far less about the ligands of group 2. With the exception of LILRA4, which binds to CD317 and is reported to regulate toll-like receptor (TLR) -7 and -9 responses in plasmacytoid dendritic cells [10], the remainder of the group 2 LILR remain unclassified, as orphan receptors.

The inhibitory LILRB3 and activating LILRA6 are examples of group 2 LILR that currently have no identified ligand. In common with most other LILR they are expressed by myelomonocytic leukocytes, but in contrast to others, they are the only ‘paired’ receptors in the LILR family, since they have almost identical extracellular domains but differ in their signalling activities [11]. A particularly interesting feature of the LILRB3 and LILRA6 genes is the high level of non-synonymous variation. The variable nucleotides exhibit similar distributions in both genes [12].

In this study we provide evidence that LILRB3 and LILRA6 interact with a novel ligand exposed on necrotic epithelial cells. We explore this interaction in the context of LILRB3 and LILRA6 allelic variation and demonstrate recognition of a ligand associated with cytokeratin 8.

## RESULTS

### LILRB3 binds to a ligand expressed by glandular epithelial cells

**Figure 1:**
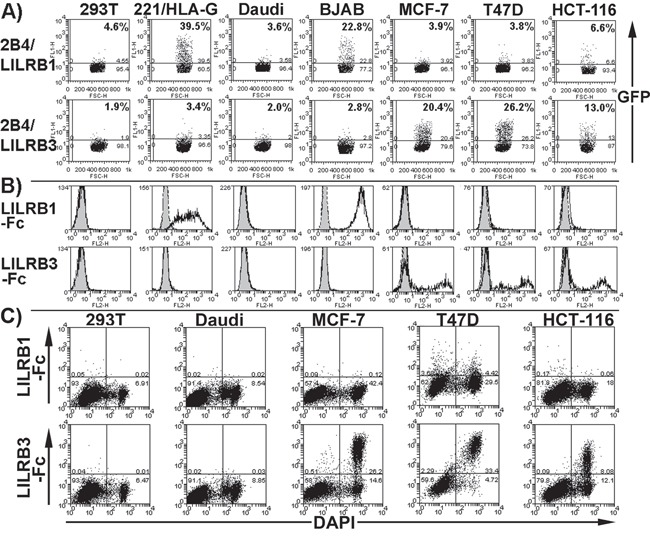
LILRB3 interacts with a ligand on glandular epithelial cells **A.** Flow cytometry dot plots showing that 2B4 reporter cells expressing hybrid proteins of LILRB1 and commonly occurring allelic variant of LILRB3 (allele LILRB3*12) and the human CD3ζ cytoplasmic domain produce GFP when activated through the hybrid receptor. Reporter cells were co-incubated with the glandular epithelial cell lines MCF-7, T47D and HCT-116, the non-epithelial cell line HEK293T (labelled 293T on figure), HLA-G transfected 721.221 (labelled 221/HLA-G) and B cell lines Daudi and BJAB. The percentage of GFP positive reporter cells is indicated. The results provided are representative of five experiments. **B.** Flow cytometry histogram plots displaying staining of cells with a LILRB1-Fc or LILRB3-Fc (allele LILRB3*12) fusion protein (no fill) or a non-hybrid Fc negative control (grey fill). Representative results of five experiments are shown. **C.** LILRB3-Fc (allele LILRB3*12), but not LILRB1-Fc, binds primarily to dead epithelial cells. Cell death was determined by the uptake of the nuclear stain DAPI. Dead cells intrinsically comprised a proportion of the cells. Representative results of four experiments are shown.

To screen for LILRB3 ligands on the surface of tumour cells, we constructed 2B4 reporter cells that expressed the extracellular domains of a commonly occurring allelic variant of LILRB3 [allele LILRB3*12, (12)], or LILRB1 fused to the human CD3ζ cytoplasmic domain. Signalling through these hybrid receptors results in the expression of GFP, which can be detected by flow cytometry. LILRB3 reporter cells were co-seeded and cultured with a wide range of human tumour cell lines including those derived from B cells and epithelial cells. Figure 1A shows results from a selection of these lines. The EBV positive and negative Burkitt's lymphoma B cell lines Daudi and BJAB, the embryonic kidney cell line HEK293T and an HLA-G transfected EBV transformed B cell line 721.221 did not induce the expression of GFP in the LILRB3 reporter 2B4 cells. In contrast, the epithelial cell line MCF-7 (breast glandular), T47D (breast ductal) and HCT-116 (colon) induced the expression of GFP, suggesting that a ligand for LILRB3 is present on these cells (Figure 1A). To demonstrate that this effect was specific to LILRB3, 2B4 cells expressing LILRB1 (which binds to MHC class I molecules) fused to the human CD3ζ cytoplasmic domain, were also co-cultured with the panel of cells. The level of GFP expression in the LILRB1 2B4 cells followed the known level of expression of MHC class I molecules on each of the target cell types. Levels of MHC class I molecules were determined by staining with the pan HLA class I mAb W6/32 (data not shown). The epithelial cell lines did not activate the LILRB1 2B4 cells to a high level (Figure 1A). Untransfected 2B4 cells were not activated by any of the cell lines tested (data not shown).

Flow cytometry of cells stained with recombinant, soluble LILRB3 (allele LILRB3*12) fused to the Fc portion of human IgG1 bound to glandular epithelial tumour cells (Figure 1B), confirming the pattern of 2B4 activation (Figure 1A). Co-staining with the DAPI nuclear stain indicated LILRB3-Fc binding to dead epithelial tumour cells (Figure 1C), suggesting that a LILRB3 ligand is exposed on these cells following necrosis. The pattern of control LILRB1-Fc staining was consistent with LILRB1-2B4 reporter cell activation (Figure 1B), and did not display any increase in binding to dead cells (Figure 1C).

Non-tumour human breast epithelial cells were then assessed using the 2B4 reporter cells. Epithelial cells cultured from four subjects tested induced expression of GFP by the LILRB3 reporter (Supplementary Figure S1), suggesting that expression of the ligand of LILRB3 is not specific to tumour cells and may also be expressed on cells of glandular epithelial origin, whether normal or transformed.

### The ligand of LILRB3 is exposed on necrotic glandular epithelial cells

The binding of LILRB3 to dead epithelial tumour cells was assessed further by staining cells with recombinant Fc fusion molecules following treatment with H_2_O_2_ or NaN_3_ (to induce necrosis), staurosporine (STS, to induce apoptosis) or subjected to mechanical damage by repeated trituration. Cell death was confirmed by Annexin V and DAPI staining (Supplementary Figure S2), while necrosis and apoptosis were determined by morphological features such as cell shrinkage (apoptosis), cellular swelling (necrosis) and formation of apoptotic bodies (Supplementary Figure S3).

**Figure 2:**
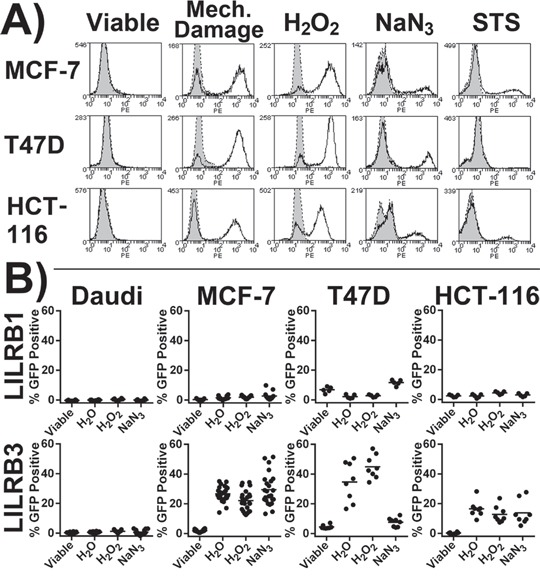
LILRB3 recognises an epitope exposed on necrotic glandular epithelial cell lines **A.** Staining of treated cells with LILRB3-Fc (allele LILRB3*12, no fill) or Fc negative control (grey fill). Cells were treated to induce lysis (H_2_O or by mechanical damage), necrosis (H_2_O_2_ or NaN_3_) or apoptosis (STS). Viable target cells underwent primary staining whilst adhered to culture plate. Representative results of 4 experiments are shown. **B.** 2B4 reporter cells were incubated with target cell lines previously treated with either H_2_O (to induce osmotic lysis), H_2_O_2_ or NaN_3_ (both necrotic agents). Viable adherent cells in lane 1 were washed to remove any dead cells prior to the addition of reporter cells. The LILRB3 2B4 reporter cells expressed the extracellular region of allele LILRB3*12. Results are from four independent experiments, each treatment was performed in duplicate. Results from an additional five independent experiments where MCF-7 cells were assessed alone are also included. Bars indicate mean values.

LILRB3-Fc (allele LILRB3*12) was not observed to bind viable cells (adherent cells were stained *in situ* prior to harvesting to avoid cell damage during cell dissociation) but bound strongly to MCF-7, T47D and HCT-116 cells following H_2_O_2_ induced necrosis and mechanically induced lysis (Figure 2A). There was moderate binding to cells treated with NaN_3_. Following STS treatment, only a small proportion of apoptotic cells were bound by LILRB3-Fc (Figure 2A). LILRB3 was not observed to bind to Daudi or 293T cells either before or following treatments (data not shown). Binding of LILRB1-Fc was not affected by the cell treatments (data not shown).

To confirm LILRB3 recognition of a ligand exposed on necrotic cells, 2B4 reporter cells were co-cultured with target cells previously treated with H_2_O_2_ or NaN_3_ and following H_2_O mediated lysis (Figure 2B). The LILRB3 reporter (allele LILRB3*12) was activated by all three epithelial cell lines following H_2_O and H_2_O_2_ treatments but not by viable cells. The LILRB3 reporter also responded to MCF-7 and HCT-116 (but not T47D) cells previously treated with NaN_3_. Both Daudi (Figure 2B) and 293T (data not shown) failed to activate the reporter irrespective of treatments. LILRB1 reporter responses were largely unaffected by the target cell treatments (Figure 2B). The 2B4 system is highly sensitive to STS induced apoptosis and was not compatible with STS treated cells.

### Polymorphism of LILRB3 influences binding to the glandular epithelial cell ligand

*LILRB3* and *LILRA6* genes display substantial polymorphic variation that results in amino acid substitutions [12]. Analysis of *LILRB3* and *LILRA6* cDNA sequences provided statistically significant evidence that variation at residues 36, 46, 97, 164, 182, 265, 318, 327, 377 and 386 of the mature protein has been subject to positive selection (Supplementary Table S1, analysis was performed using sequences provided in Supplementary Table S2 Residues 36 and 97 align to positions known to make up the MHC class I molecule- binding sites of the group 1 LILR proteins, along with polymorphic sites 38, 67, 99 and 126 [8, 13]. To determine whether these and any other amino acids are similarly involved in the binding of LILRB3 and LILRA6 to glandular epithelial cells, constructs of selected LILRB3 and LILRA6 variants were prepared.

**Figure 3:**
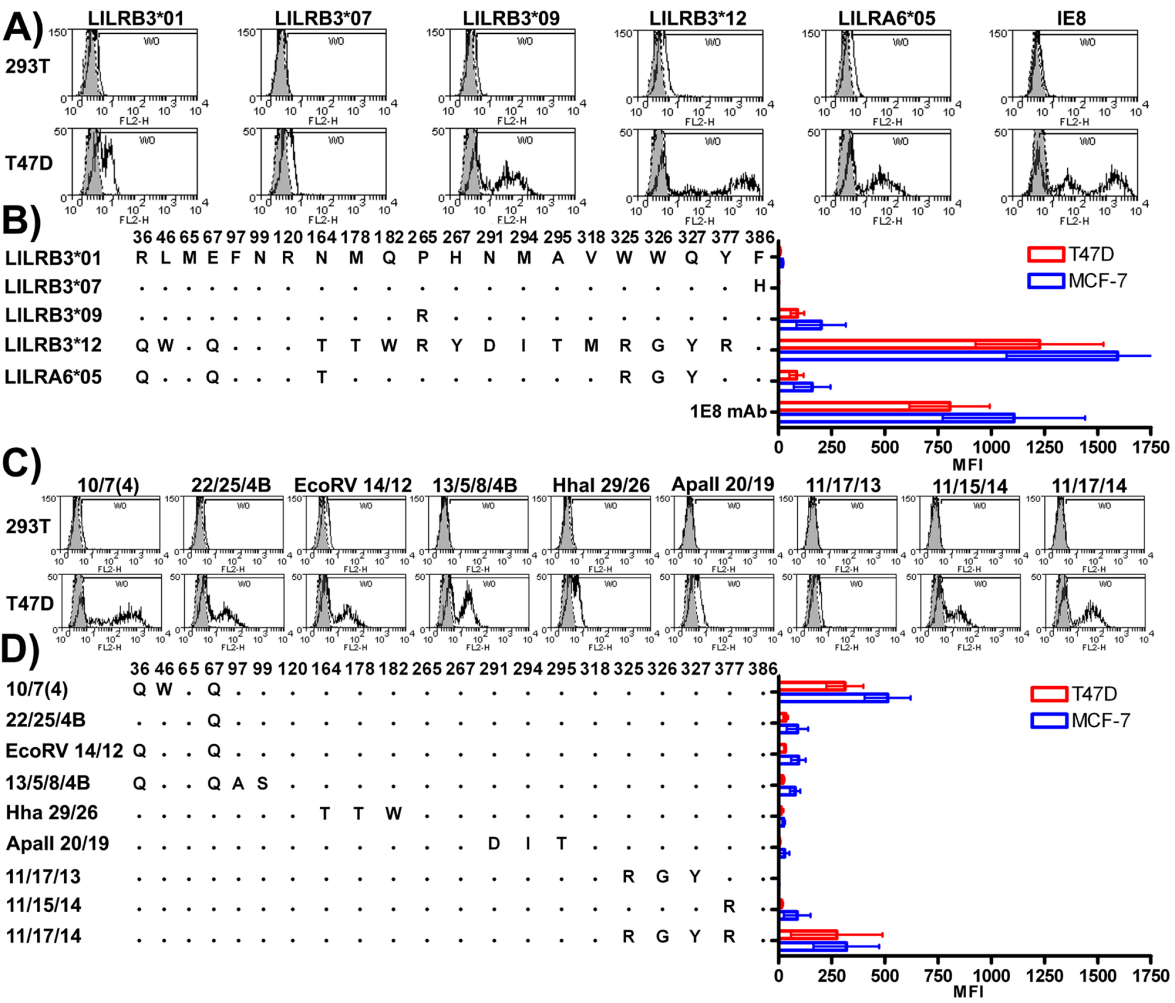
LILRB3-Fc and LILRA6-Fc polymorphic variants differentially bind to mechanically damaged glandular epithelial tumour cells lines **A.** The non-epithelial HEK-293T and the epithelial tumour cell T47D were stained with naturally occurring variants of LILRB3-Fc and LILRA6-Fc. Representative histograms are shown; shaded peaks indicate staining with the Fc negative control protein. Cells were stained with the anti-human cytokeratin 8-specific monoclonal antibody 1E8 as a positive control. **B.** The overall mean average and standard deviation resulting from four replicate experiments where each treatment was performed in duplicate are provided. Individual LILRB3-Fc and LILRA6-Fc mean fluorescence intensity (MFI) values were normalised for background by subtracting the Fc negative control MFI values. Representative staining with chimeric Fc molecules that combined motifs from high and low ligand binding LILR variants are provided in **C.** while the overall mean average and standard deviation resulting from four replicate experiments are shown in panel **D.**

**Figure 4:**
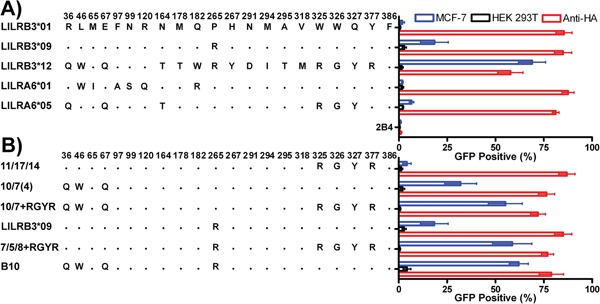
LILRB3 and -A6 polymorphisms influence cellular recognition of mechanically damaged breast cancer cells Parental 2B4 reporter cells (2B4), and 2B4 cells transfected with the naturally occurring LILRB3 and LILRA6 variants **A.** and chimeric LILRB3/A6 sequences **B.** were used in co-culture with epithelial MCF-7 (striped bars) and non-epithelial HEK-293T (white bars) target cells. Mean and standard deviation values from 6 replicate experiments are shown. LILRB3/A6 molecules were cross-linked with a monoclonal antibody specific for the HA tag introduced into the N-terminus of the LILR during construction.

An initial screen of the LILR-Fc fusion proteins for their binding to mechanically damaged epithelial cell lines identified two products from the alleles LILRB3*01 and LILRB3*12 that displayed very low, and very high, binding respectively (Figures 3A&3B), while products from alleles LILRB3*09 and LILRA6*05 exhibited intermediate binding. Similar results were found in 2B4 reporter assays (Figure 4A).

Sequences were reciprocally exchanged between the low binding LILRB3*01 and high binding LILRB3*12 followed by the expression of the hybrid LILRB3 molecules as LILR-Fc fusion proteins. They were used to stain MCF-7 cells. These experiments identified three broad regions in Ig domains D1, D3 and D4 that appeared to cooperate in binding (Supplementary Figure S4). Amino acids associated with ligand binding included Q36, L46 and Q67 in Ig domain D1, R265 and Y267 in Ig domain D2, and M318, R325, G326, Y327 and R377 in Ig domain D4.

To refine the LILRB3 ligand binding sites further, these data were used to design a higher resolution screen of the amino acids identified in the hybrid LILRB3. This panel of chimeric LILRB3-Fc fusion molecules was used to determine which of the amino acids in each of the broad regions identified in Supplementary Figure S4 were most important for binding (Figures 3C&3D). The LILRB3 amino acids most closely associated with binding to the ligand were: Q67, which was enhanced by W46 (D1 domain); R265 (D3 domain); R325/G326 and Y327 (encoded by linked single nucleotide polymorphisms [SNPs]) when in the presence of R377 (D4 domain) (Figures 3A-D).

2B4 reporter cells, transfected with the naturally occurring LILRB3 and LILRA6 variants alongside chimeric LILRB3 molecules confirmed that binding is influenced by more than one linear motif and supported the earlier findings for binding sites in D1 and D3 when LILR-Fc fusion proteins were used to stain MCF-7 cells (Figures 4A&4B). There was, however, a discrepancy regarding the RGYR motif in the D4 domain. With soluble LILRB3-Fc fusion proteins, this motif, when incorporated into the non-ligand binding LILRB3*01 allele, showed moderate binding to the LILRB3 epithelial ligand (Figures 3C&3D). When this chimeric LILRB3 was expressed as a receptor on 2B4 cells, however, the motif failed to induce a response unless in the presence of either of the D1 or D3 binding-associated motifs (Figure 4B).

### Interaction with the glandular epithelial cell ligand of LILRB3 involves more than one Ig domain

Data from binding and reporter assays were used to position the identified ligand- binding sites on molecular models of LILRB3 based on the structures of related molecules. Sites within the D1 domain of LILRB3 (residues 46 and 67) were in similar positions on structures based on LILR templates (Supplementary Figure S5). The placement of residue 46 in these models differed considerably from that of the structure based on the more distantly related NKp46 (structure 1p6f:A). The predicted structures of the D3 and D4 Ig domains of LILRB3 were produced from template alignments featuring comparable regions within LILRB1, LILRB2, LILRB4 and the more distantly related KIR molecules. Sites identified as interacting with the glandular epithelial ligand within D3 [265(R)] and D4 [325-327 (RGY) and 377(R)] occurred in similar positions in all 4 models (Supplementary Figure S6). All models are consistent with 325-327 and 377 being in close proximity on the surface of LILRB3, although separated by ∼50 amino acids.

**Figure 5:**
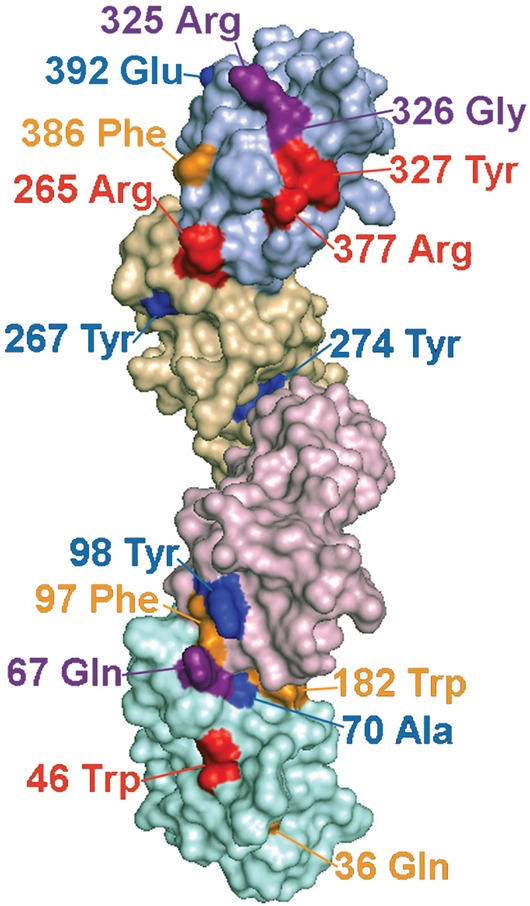
Predicted locations of LILRB3 and LILRA6 polymorphisms that alter binding to necrotic glandular epithelial cells, and sites under strong evolutionary selection pressure Molecular model of LILRB3 based on the structure of related LILR proteins. Ig domains are shaded in different colours with D1 at the bottom of the figure and D4 at the top. The labelled polymorphic residues are coloured to indicate their influence on ligand binding and selection pressure, as follows: Red= Polymorphic amino acids implicated in ligand binding that have undergone significant positive selection pressure; Purple= Putative ligand binding amino acids that are not under significant positive selection; Yellow=Amino acids that are undergoing positive selection, but do not appear to influence ligand binding; Blue=Amino acids undergoing negative selection. Amino acid 326 (coloured purple) is under significant negative selection and also implicated in binding.

A four Ig domain structure of LILRB3 was constructed from the Raptor X generated models of D1-D2 and D3-D4 (Figure 5), both of which scored well in terms of energy and stereochemical properties (Supplementary Tables S3 and S4), with 95.5% and 94.4% of residues occurring within their most favoured positions respectively. The sites identified as interacting with the glandular epithelial ligand were predicted to occur on one face of the LILR molecule (Figure 5).

### LILRB3 interacts with a cytokeratin - associated ligand

**Figure 6:**
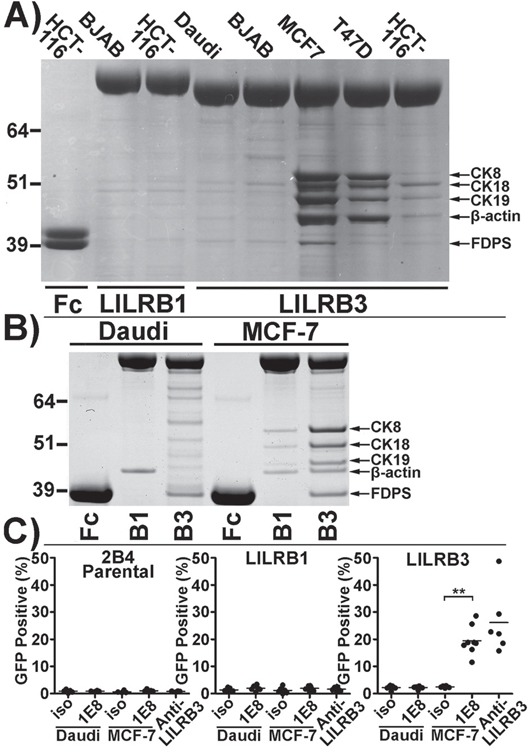
The LILRB3 ligand co-purifies with cytokeratin 8 from the lysates of glandular epithelial cell lines **A.** Coomassie blue stained SDS-PAGE gel of proteins immunoprecipitated by LILRB3*12-Fc from membrane-rich lysates of the epithelial cell lines MCF-7, T47D, and HCT116 compared with the non-epithelial cell lines BJAB and Daudi. The high molecular weight molecules (>64 kDa) in lanes 2-7 are LILR-Fc proteins. The Fc negative control protein in lane 1 has an apparent molecular weight of ∼40kDa. **B.** A repeat Coomassie stained SDS-PAGE gel of proteins immunopreciptated by LILRB3*12-Fc from Daudi and MCF-7 cells. Bands were excised from the gel and were identified by mass spectrometry as cytokeratins (CK) 8, 18 and 19, β-actin and farnesyl diphosphate synthase (FDPS). **C.** Isolation of ligand of LILRB3*12 from cell lysates with the anti-human cytokeratin 8 specific mAb 1E8: Ligands captured from MCF-7 lysates (but not from Daudi B cells) on 1E8-coated microsphere beads were cultured with 2B4 reporter cells. Beads coated with isotype control antibody (iso) prior to incubation with lysates were used as a negative control. Beads coated in an anti-LILRB3 antibody were used as a positive control. The results shown are from 4 replicate experiments. Bars indicate mean values. Statistical significance was assessed using a two-tailed Mann Whitney test (_**_p<0.001).

LILRB3*12 displayed the strongest binding to necrotic glandular epithelial cells. Products of this allele were used subsequently to identify the ligand. The LILRB3*12-Fc fusion protein was used to immunoprecipitate the putative LILRB3 ligand from lysates of T47D, MCF7 and HCT116 epithelial cells, using the non-epithelial B cell lines Daudi and BJAB as controls (Figure 6A). On SDS-PAGE gels, several reproducible, immunoprecipitated bands were identified in samples from the T47D, MCF7 and HCT116 epithelial cell lines that had also activated the LILRB3*12 2B4 reporter cells and had stained with LILRB3*12-Fc. In cells that neither activated the LILRB3*12 2B4 reporter cells nor stained with LILRB3*12-Fc, only low levels of background bands were seen.

Following analysis of the major bands by mass spectrometry (Figure 6B), five main proteins were identified. Three of these were cytokeratins 8, 18 and 19, all of which are cytoskeletal intermediate filament proteins associated with glandular, ductal epithelial cells. The remaining proteins were the cytoskeletal component β-actin, which was isolated solely from epithelial cell lysates, and farnesyl diphosphate synthase (FDPS), an enzyme that may be involved in the metabolism of the human Vγ9Vδ2 T cell phosphoantigen, isopentenyl pyrophosphate [14]. FDPS was isolated from both epithelial and non-epithelial cell lysates alike. LILRB1-Fc also immunoprecipitated weak protein bands containing cytokeratins 8, 18 and 19 from MCF-7 cell lysates, and β-actin from both MCF-7 and Daudi cell lysates. Despite the apparent ‘stickiness’ of these intracellular cytoskeletal elements, the strong interaction of LILRB3*12 with the glandular ductal epithelial cytokeratins was investigated further. FDPS was ruled out as a potential ligand of LILRB3 as its expression could not be detected by flow cytometry (data not shown).

To confirm that LILRB3*12 binds to a complex of proteins involving cytokeratin 8 and that this binding was responsible for activating the LILRB3*12 2B4 cells, microsphere beads coated with the anti-human cytokeratin 8 specific monoclonal antibody 1E8, or an isotype control, were incubated with membrane-enriched lysates from MCF7 or Daudi B cells prior to co-incubation with 2B4 reporter cells expressing either LILRB3*12 or LILRB1, or with parental 2B4 cells. Ligands captured from the MCF-7 lysate by 1E8, but not the isotype control, activated the LILRB3*12 - expressing 2B4 cells to produce GFP (Figure 6C); a similar percentage of LILRB3*12 2B4 cells were activated by a cross-linking anti-LILRB3 antibody. The parental 2B4 cells and the LILRB1 expressing 2B4 cells were not activated by 1E8 ligands captured from either the MCF-7 or the Daudi cells. An anti-LILRB3 antibody also failed to activate these cells.

These results are consistent with the finding that proteins affinity purified from epithelial cells along with cytokeratin 8 associate with LILRB3*12.

### The LILRB3 ligand requires expression of the cytokeratin 8 gene

**Figure 7:**
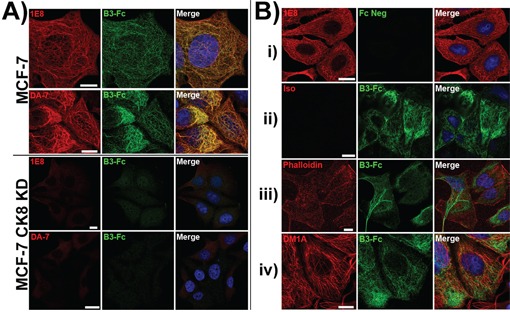
LILRB3-Fc binding of MCF-7 cells following shRNA silencing of the expression of cytokeratin 8 as assessed by confocal microscopy **A.** LILRB3*12-Fc binding colocalised with the binding of the mAbs 1E8 (anti-cytokeratin 8) and DA-7 (anti-cytokeratin 18) in permeablised MCF-7 cells that were previously transduced with a non gene-specific shRNA construct. shRNA constructs were used to stably silence the expression of cytokeratin 8 in MCF-7 epithelial cells. Binding of both mAbs and LILRB3*12-Fc were greatly reduced in MCF-7 cytokeratin 8 knock down cells (CK8 KD). Bar represents 10μm. **B.** MCF-7 cells that were previously transduced with a non gene-specific shRNA construct were stained with either: i) the 1E8 mAb and Fc negative control construct, or ii) isotype control mAb and the LILRB3*12 Fc fusion. Both the isotype and control Fc displayed minimal binding. LILRB3*12-Fc staining did not co-localise with phalloidin staining (actin, panel iii) or DM1A mAb (anti-tubulin, panel iv).

To further clarify the link between the expression of cytokeratin 8 and the expression of the ligand for LILRB3*12, shRNA constructs were used to stably silence the expression of cytokeratin 8 in MCF-7 epithelial cells. Using confocal microscopy, the intracellular levels of the 1E8 cytokeratin 8 epitope and LILRB3*12-Fc ligand were compared in MCF-7 cytokeratin 8 knock-down cells (MCF-7 CK8 KD) and MCF-7 cells that had been transduced with a non gene-specific shRNA construct (Figure 7). Compared with the control cells, which showed normal 1E8 and LILRB3*12-Fc binding (with clear co-localisation), MCF-7 CK8 KD cells expressed only background levels of the 1E8 cytokeratin 8 epitope and binding of LILRB3*12-Fc. The expression level of cytokeratin 18, as determined by the binding of DA-7, was also greatly reduced in MCF-7 CK8 KD cells (Figure 7), demonstrating that cytokeratin 18 requires cytokeratin 8 for stable expression [15]. Expression of cytokeratin 19, the other binding partner of cytokeratin 8, was also greatly reduced following the silencing of cytokeratin 8 expression (data not shown).

**Figure 8:**
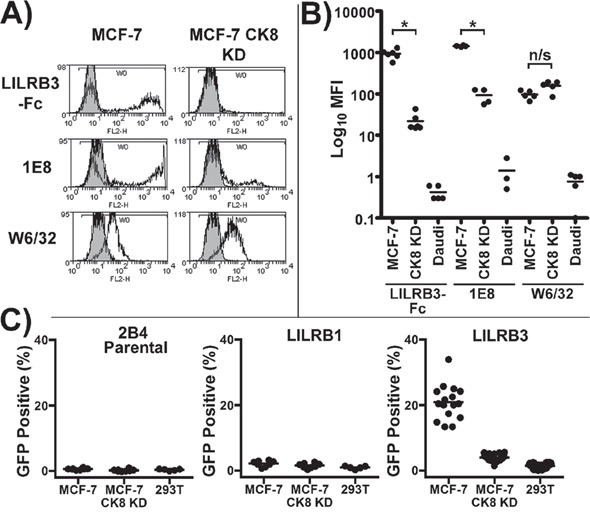
LILRB3 binding is dependent upon expression of cytokeratin 8 **A.** LILRB3*12-Fc binding of mechanically damaged MCF-7 cells following shRNA silencing of cytokeratin 8 expression. The levels of the 1E8 cytokeratin 8 epitope, LILRB3*12-Fc and MHC class I (W6/32) were compared in MCF-7 cytokeratin 8 knock down cells (MCF-7 CK8 KD) and MCF-7 cells transduced with a non gene-specific shRNA construct. **B.** Analysis of the mean fluorescence intensity of the 1E8 epitope, the LILRB3 binding ligand and MHC class I on MCF-7, MCF-7 CK8 KD and Daudi B cells. The binding of LILRB3*12-Fc and 1E8 was significantly reduced on mechanically damaged MCF-7 CK8 KD cells in comparison to MCF-7 cells, whilst both displayed similar expression levels of MHC class I. Bars indicate mean values (_*_p<0.01 using a two-tailed Mann Whitney test; ns= not significant). **C.** Activation of LILRB3 2B4 reporter cells by necrotic MCF-7 cells following shRNA silencing of the expression of cytokeratin 8. Target cells were treated with H_2_O_2_ to induce necrosis prior to incubation with reporter cells. MCF-7 cells strongly activated LILRB3*12 expressing 2B4 cells whilst the MCF-7 CK8 KD cells activated ∼4% LILRB3*12 2B4 cells. HEK293T non-epithelial cells were used as a control. The results from 4 replicate experiments are shown. For each experiment, assessment of LILRB1 and parental 2B4 reporter cells was performed in duplicate, while assessment of LILRB3*12 was performed in quadruplicate. Bars indicate mean values.

Analysis of the mean fluorescence intensity of the 1E8 epitope, the LILRB3*12-binding ligand and MHC class I on mechanically damaged MCF-7 and MCF-7 CK8 KD cells showed that the expression of the LILRB3*12 ligand, but not MHC class I, was significantly different between the MCF-7 and MCF-7 CK8 KD cells (Figure 8A&8B). LILRB3 binding was assessed in this set of experiments using the LILRB3*12-Fc fusion protein.

Finally, necrotic MCF-7 CK8 KD cells previously treated with H_2_O_2_ were used with control MCF-7 and HEK293T non-epithelial cells in a co-culture assay with LILRB3*12, LILRB1 and non-LILR expressing parental 2B4 reporter cells. None of the cell lines activated the parental 2B4 cells or the LILRB1 expressing cells. The LILRB3*12-expressing 2B4 cells were strongly activated by MCF-7 cells whilst the MCF-7 CK8 KD cells activated a low percentage of LILRB3*12 2B4 cells (Figure 8C). Similar results were obtained using mechanically damaged target cells and targets treated with NaN_3_ (data not shown). The recognition of MCF-7 cells by 2B4 reporter cells expressing LILRB3*09 and LILRA6*05 was also greatly reduced following the silencing of cytokeratin 8 expression (Supplementary Figure S7). These shRNA knock-down experiments confirm that the expression of the LILRB3 ligand is dependent upon the expression of the cytokeratin 8 gene. This series of experiments are all consistent with the assertion that certain allelic variants of LILRB3/A6 interact with a ligand, or ligands, associated with the expression of cytokeratin 8 by glandular epithelial cells.

## DISCUSSION

We provide evidence that certain allelic variants of LILRB3 and LILRA6 recognise a ligand from necrotic glandular epithelial cells that is associated with cytokeratin 8 and is displayed on the cell-surface. The precise identity of the ligand remains to be determined. Cytokeratins are conventionally known as intracellular cytoskeletal molecules that form intermediate filaments (IF) in epithelial cells. Cytokeratin 8 forms heterodimers with cytokeratin 18 or 19 which then heteropolymerise to form intermediate filaments. These in turn interact with a wide range of other intracellular proteins, some of which may co-purify with cytokeratin 8. We showed that knock-down of cytokeratin 8 ablated the binding of LILRB3*12 to epithelial cells. However, intermediate filament polymerisation, and association with other proteins is dependent on the presence of cytokeratin 8. Therefore, the loss of the ligand on cytokeratin 8 knock-down cells could be due either to the ablation of direct binding to the cytokeratin itself, or indirectly due to the loss of a protein associated with cytokeratin 8. For this reason, we cannot unequivocally identify cytokeratin 8 as the ligand.

Given the intracellular location of cytokeratin 8 our findings were unexpected. There are several reports of cytokeratin epitopes being present on the surface of epithelial tumours and cell lines [16-23]. However, we found little evidence for the presence of LILRB3 and LILRA6 ligand on viable cells. An explanation might be that normal cultures generally contain a proportion of necrotic cells, which are responsible for exposure of cytokeratin. Another possibility concerns sticking of cell debris, containing cytokeratin, to the surface of live cells. Some epithelial tumour cells release measurable levels of cytokeratin into plasma after they die by either necrosis or apoptosis. Levels of cytokeratin in plasma have been proposed, therefore, as a biomarker to predict and monitor responses to chemotherapy [24]. LILRB3 2B4 reporter cells did not respond to soluble cytokeratin released from necrotic cells in tissue culture medium (data not shown). Furthermore, there was no evidence that etoposide-induced apoptosis, in contrast to necrosis, induces the exposure of the LILRB3 ligand on any cell line tested (Figure 2A). This difference may be explained partially by the cleavage and reorganisation of cytokeratin 8/18 intermediate filaments during programed cell death, a process mediated by caspases, that does not occur in necrosis [25].

The concept that transformed, stressed and damaged cells expose intracellular molecules that activate components of the innate immune system has been proposed before [26]. For example, there is evidence that myeloid C-type lectin receptors, including DNGR-1 and DEC205, sense damaged self [27]. It is proposed that sterile inflammation may be the consequence of recognition of damage-associated molecular patterns, or DAMPS [28]. Recognition of DAMPs may lead to initiation of T cell responses. Those DAMPs that have been characterised comprise intracellular contents that have been exposed upon tissue damage, such as F-actin [28]. Indeed it has been proposed that exposure of the cytoskeleton is an evolutionarily conserved signal of damage that excites innate immunity.

Tumour cell necrosis is a common feature of malignancy, often occurring within the centre of rapidly growing tumours. Our data suggest that the process of necrosis leads to exposure of the cytokeratin-associated ligand which is then available for recognition by paired activating and inhibitory LILRB3 and LILRA6 receptors expressed by dendritic and other myelomonocytic cells. They are consistent with recognition of cytokeratin ligand not being restricted to tumour cells. It is possible that macrophage interaction with cytokeratin, through LILR, is a signal for recognition of glandular epithelial necrosis, which is marked in tumours as they generally contain a high proportion of necrotic cells. It is worth noting that there are other situations of pronounced necrosis, such as liver disease [29]. The balance of signals transmitted by inhibitory LILRB3 and activating LILRA6 might therefore determine whether an immune response is initiated to the dead tumour cell, thereby to influence the wider immune response within the tumour microenvironment. In this regard it is not surprising that cytokeratin behaves as a DAMP. However, our finding that exposed cytokeratin may interact both with inhibitory and activating receptors is not consistent with simple activation but, rather with some degree of regulation of response.

Polymorphic variation in cell surface molecules that affect innate immune regulation is generally considered to occur in response to immune escape by pathogens. An interpretation of our data is that the variation is an evolutionary response to pathogens that influence, or down-regulate, inflammatory mechanisms to gain a selective advantage. Another possibility is that the polymorphism has been driven by immune response to cell transformation. The finding that polymorphic variants of LILRB3 and LILRA6 have very different ligand binding characteristics suggests that cells carrying these receptors, in individuals possessing high or low ligand binding variants of inhibitory LILRB3 or activating LILRA6, will be variably activated by epithelial tumours. The scope for variability in the response to the cytokeratin - associated ligand becomes more complex when combinations of high and low binding variants of the activating and inhibitory receptors are considered alongside evidence of copy number variation in the *LILR* genes [12, 30]. There are other examples of paired activating and inhibitory receptors, including the *KIR*, whose genes are located near to the *LILR* loci in the neighbouring region of chromosome 19 [11, 31-33].

In common with certain *KIR*, *LILRB3* and *LILRA6* display the hallmark of genes undergoing positive selection, with several substitutions displaying statistically significant evidence of positive selection pressure (Supplementary Table S1). Four polymorphic amino acids are located in motifs in the D1, D3 and D4 Ig domains that we have shown influence the recognition of tumour cells by these LILR receptors (Figure 5).

There are no other data on the physiological role of LILRB3 and LILRA6. The differential binding characteristics of activating and inhibitory variants to necrotic tumour cells could shape local inflammatory responses to epithelial tumours. An alternative explanation of our findings is that LILRB3/A6 binding is a non-physiological artefact due to exposure of molecules normally confined to the cytoplasm. This remains a possibility, however most tumours contain a core of necrotic cells which potentially could interact with macrophages. The LILRB3/A6 interaction with these cells may be exploitable in an anti-tumour immunotherapy.

## MATERIALS AND METHODS

### Immunoprecipitation

Immunoprecipitation was performed using protein A Dynabeads (Life Technologies) coated with LILR-Fc protein. 50μl PBS-T-washed Dynabeads were loaded with LILR-Fc following incubation with 50μl of 250μg/ml freshly made LILR-Fc at 4°C for 1 hour followed by two washes with PBS-T. The construction and preparation of the LILR-Fc protein is described in the Supplementary Material.

Cell lysates (100μl) were precleared by incubation at 4°C for 1 hour with 25μl protein A Dynabeads. 50μl of LILR-Fc loaded Dynabeads were then added to the precleared lysate for 1hr at 4°C. Beads were washed thrice in cold PBS-T and resuspended with LDS loading buffer (30mM Glycine pH2.8, 1x Nupage LDS buffer and reducing agent [Life Technologies]). Samples were denatured at 70°C for 10 min, before loading onto a Nupage 4-12% Bis-Tris SDS PAGE gel (Life Technologies) alongside SeeBlue II marker (Life Technologies). Gels were run in MOPs buffer (Life Technologies) for 45 minutes at 200V. Protein bands were visualised following Coomassie Blue staining.

Protein bands in gels were identified by fingerprinting of tryptic peptide using an Applied Biosystems 4800 Plus MALDI-TOF-TOF Mass Spectrometer in CIMR/IMS Proteomics Facility (CIPF) of the Cambridge Institute for Medical Research.

### Preparation of cytokeratin 8 coupled beads

Sphero™ 5.0-5.9 μm diameter, carboxyl pink fluorescent beads (10mg), (Saxon Europe, Kelso UK) were conjugated in 2ml of 20μM sodium acetate buffer pH5.0 with 0.3mg of protein A (Sigma-Aldrich, St. Louis, MO, USA), using 7.65mM N-(3-Dimethylaminopropyl)-N′-ethylcarbodiimide hydrochloride as a crosslinking agent (Sigma-Aldrich), for 2hrs at RT. Following 2 washes with PBS pH7.4, 2mg of protein A-conjugated beads were incubated for 1hr at room temperature with one of the following: 45μg of the anti-human cytokeratin 8 monoclonal antibody 1E8 (Santa Cruz Biotechnology, Dallas, TX, USA), anti-human LILRB3, (clone 222821, R&D Systems) or an IgG2a isotype control, (clone 20102, R&D Systems). Following 2 washes with PBS pH7.4, 1mg of antibody coated beads was incubated at 4°C for 1hr with either 500μl MCF-7 or Daudi membrane-enriched cell lysates (prepared as described above). Beads were washed twice in PBS + 0.02% Tween, and resuspended in PBS pH7.4, 5mM EDTA, 2% BSA, 0.02% sodium azide and 1x Proteoblock protease inhibitor cocktail (Fermentas) and stored at 4°C. Beads were washed with PBS and sonicated prior to use.

### Flow cytometry

The following mouse monoclonal antibodies (mAb) were used in flow cytometry: W6/32, anti-human HLA-Class I; 1E8, anti-human cytokeratin 8 (Santa Cruz Biotechnology); DA-7, anti-human cytokeratin 18 (Biolegend); BA-17 anti-human cytokeratin 19, (R&D systems and an IgG2a isotype control (Sigma-Aldrich). The rabbit monoclonal antibody EPR4628, anti-human farnesly diphosphate synthase (FDPS) (GeneTex, Irvine, CA, USA), was used alongside negative control rabbit IgG (Sigma-Aldrich).

All primary antibodies were used at a concentration of 20μg/ml, while LILR-Fc fusion molecules where used at 60μg/ml, unless otherwise stated. Primary staining of adherent cells was performed following detachment using NECDB unless otherwise stated; staining was also performed *in situ* on 6 well tissue culture plates before detachment and subsequent secondary staining. In both cases, 10^5^ cells were stained in PBS pH 7.4, 5% FBS for 45 minutes on ice followed by two washes in PBS pH 7.4, 5% FBS. All secondary staining incubations were performed on ice for 45 minutes with one of the following, where appropriate: 100μl/ml rabbit F(ab’)_2_ anti-mouse IgG-RPE (Serotec, Kidlington, UK); 2μg/ml swine anti-rabbit IgG-FITC (Dako); or 1.25μg/ml goat F(ab’)_2_ anti-human IgG Fcγ-RPE (Jackson ImmunoResearch Laboratories, West Grove, PA, USA) for cells stained with LILR-Fc proteins. Cells were washed twice with PBS pH 7.4, 5% FBS followed by a final wash with PBS prior to acquisition on a FACScan (BD Biosciences, Franklin Lakes, NJ, USA) or CyAn ADP (Beckman Coulter) flow cytometers. Additionally, cell viability was assessed by addition of 25μg/ml DAPI di-lactate (Life Technologies) 2 min before acquisition. Analysis was performed using Cell Quest (BD Biosciences) or Weasel 3.0 (http://www.wehi.edu.au).

Externalization of phosphatidylserine, an indicator of apoptosis, was assessed by Annexin V-APC staining (BD Bioscience) following the manufacturer's recommended protocol and acquired using a CyAn ADP flow cytometer.

### Confocal microscopy

MCF-7 cells were seeded on uncoated glass coverslips and left overnight to adhere. The following cell preparations and staining were performed on ice: Cells were fixed with 4% paraformaldehyde in 250mM Hepes buffer for 10 mins and permeablised using 0.1% Triton X/PBS for 10 min before washing twice with PBS. Intracellular primary staining was performed using 20μg/ml of the mAbs 1E8, DA-7, BA-17, or 1μg/ml of DM1A (mouse anti-α-tubulin, Millipore) and 5U/ml of Phalloidin Alexa Fluor 647 (Molecular Probes) in conjunction with 60μg/ml of LILRB3-Fc fusion. Cells were stained in PBS/5% FBS for 1 hour followed by two washes with PBS/5% FBS and a further 1hr incubation with 2μg/ml goat anti-mouse Alexa Fluor 647 (molecular probes) and 1.7μg/ml goat F(ab’)_2_ anti-human IgG Alexa Fluor 488 (Jackson ImmunoResearch Laboratories), where appropriate. Cells were washed twice with PBS/5% FBS, followed by one wash with PBS. Finally, coverslips were mounted on glass slides using Mowiol 4-88 containing 0.5μg/ml DAPI. Cells were viewed using an Axio Observer Z1 confocal microscope (Ziess) with a 100x oil-immersion objective lens and images acquired using ZEN software (Ziess).

### LILR-2B4 reporter assays

The construction of the LILR-2B4 reporter cells is described in Supplementary material. Cloned or sorted (but polyclonal) 2B4 reporter cells (2×10^4^) were co-cultured at a ratio of 1:3 with target cells or 16.7μg of antigen-coated beads in 200μl RPMI/10% FBS in 96 well U-bottomed cell culture plates (Nalge Nunc, Penfield, NY, USA). Adherent target cells were either co-seeded with the 2B4 reporter cells or allowed to adhere onto the plates prior to washing (to remove dead cells) and the addition of reporter cells. Plates were centrifuged for 1 min at 200g and incubated for 20 hours at 37°C in 5% CO_2_ air atmosphere. To provide a positive control, LILR were cross-linked with either 25μl/ml anti-HA mAb (Miltenyi Biotec) followed by 25μl/ml rabbit F(ab’)_2_ anti-mouse IgG (Serotec), or 12.5 μg/ml anti-LILRB3 mAb (R&D Systems; clone 222821). The percentage of 2B4 reporter cells expressing GFP was assessed using a FACScan flow cytometer (BD Biosciences). The LILRB3 2B4 reporter cell-line expressing allele LILRB3*12 was used in all assays unless otherwise stated.

### shRNA silencing of cytokeratin 8 expression by MCF-7 cells

A replication incompetent lentiviral vector was used to stably transduce MCF-7 cells with a short mRNA hairpin (shRNA) construct designed to target human cytokeratin 8 mRNA. Transduction with an shRNA sequence with no target in the human genome was used as a negative control. Appropriate hairpin sequences were obtained from the RNAi consortium (http://www.broadinstitute.org/rnai/public/). The cytokeratin 8 specific hairpin was constructed from the oligomer pair CCGGGGATGCAGAACATGAGTATTCC TCGAGGAATACTCATGTTCTGCATCCTTT TTG and AATTCAAAAAGGATGCAGAACATGAGTA TTCCTCGAGGAATACTCATGTTCTGCATCC (TRCN0000421337), and the control hairpin from CCGGGCTTCAAGTGGGAGCGCGTGACTCGA GTCACGCGCTCCCACTTGAAGCTTTTTG and AATTCAAAAAGCTTCAAGTGGGAGCGCG TGACTCGAGTCACGCGCTCCCACTTGAAGC (TRCN0000072208).

### Cell treatments

Necrosis was induced in cell lines by the addition of 160mM NaN_3_ or 0.5mM H_2_O_2_ into the culture medium. 1μM staurosporine (STS) was used to induce apoptosis. Both treatments were performed for 16hrs. Cells were also incubated for 1 hour with H_2_O to induce osmotic lysis, or subjected to mechanical damage by repeated trituration.

### Statistical analysis

LILR-Fc binding and 2B4 reporter responses were assessed for statistical significance using a two-tailed Mann Whitney test in Graphpad 4 software (San Diego, CA, USA).

### Protein structural modelling of LILRB3 Ig domains

Models for three protein sequences encompassing D1 to D2, D2 to D3 and D3 to D4 of LILRB3*12 were generated by the structural protein prediction server Raptor X [34] using default settings. Raptor X performed well in our evaluation of online structural prediction servers (data not shown). A four Ig-domain representation of LILRB3 was constructed from the D1-D2 and D3-D4 models generated by Raptor X. Alignment of these two regions was achieved using the D2-D3 model as a structural template. All models were evaluated in terms of energy and stereochemical properties using Qmean6 [35, 36], Dfire [37] and Procheck (Ramachandran and G-factor analysis) [38]. Models were visualised using PyMol.

## SUPPLEMENTARY DATA TABLES AND FIGURES


