# Supplementary material for: Allele-specific recognition by LILRB3 and LILRA6 of a cytokeratin 8 - associated ligand on necrotic glandular epithelial cells
Source: Oncotarget. 2016 Jan 13;7(13):15618–31. doi: 10.18632/oncotarget.6905 (PMC4941265; doi:10.18632/oncotarget.6905)
Supplement: Supplementary file 1 [file oncotarget-07-15618-s001.pdf]

SUPPLEMENTARY DATA

Production of LILR-Fc DNA constructs and protein

Human macrophage RNA was extracted and cDNA synthesised as described previously [1]. Full-length *LILRB3* and *LILRA6* transcripts were amplified from cDNA using the primers: NK1078 (GCAGAGCGCGGGGTCAC) and NK1091 (TTAGTCATCTTTGAGTCAGGTGAG) for *LILRB3* and NK1078 and NK636 (GAACACCAGGACCAAGCCT) for *LILRA6*. PCRs were run in a MJ Research PTC-200 thermal cycler (Reno, NV, USA) using Phusion polymerase (Finnzymes, Vantaa, Finland) under the following conditions: 30 s at 98°C followed by 35 cycles of 98°C for 10 s, 68°C for 30 s, and 72°C for 60 s. PCR products were cloned into the TOPO vector (Life Technologies, Carlsbad, CA, USA) following the manufacturer's protocol. Fused LILR-Fc constructs were made by amplifying the extracellular sequences of the cloned *LILRB3* and *LILRA6* alleles using the primers NK1324 (ATGCAAGCTTCCCTTCCCCAAACCCAC) and NK1194 (ATGCTCTAGAGTATCTTCCCAGACCAGGT). PCR products were ligated into the Signal plgplus vector (R&D Systems, Abingdon, UK) in frame with the vector human IgG1-Fc sequence using the HindIII and XbaI sites.

Inter-allele chimeric LILRB3-Fc and LILRA6-Fc were constructed by exploitation of the natural EcoRV, HhaI, ApaII and BglII restriction sites within the *LILRB3* and *LILRA6* sequences. The sequences of all constructs were verified by cycle sequencing using BigDye Terminator v 3.1 (Applied Biosystems, Foster City, CA, USA) and an Applied Biosystems 3730xl DNA analyser.

The LILRB1-Fc construct was produced as described previously [2] and the transfection and production of LILR-Fc fusion proteins from all constructs were produced as previously described [2]. The LILRB3-Fc protein encoded by allele *LILRB3*12* [3] was used in all assays unless otherwise stated.

LILR-CD3ζ constructs and NFAT-GFP reporter cells

The extracellular regions of *LILRB3* and *LILRA6* were amplified from previously cloned sequences using the primers NK1320 (ATGCAGATCTCCCTTCCCCAAACCCAC) and NK1321 (ATGCGTCGACAGGTGTGGAGGGCGGCC). *LILR* PCR products were ligated into the pDISPLAY vector (Life Technologies) such that they were fused to the vector N terminal of the haemagglutinin A (HA) epitope and the transmembrane domain of platelet-derived growth factor receptor (PDGFR). The extracellular region of *LILRB1* was cloned into pDISPLAY using the primers NK1306 (ATGCAGATCTCACCTCCCCAAGCCCACC) and NK1309 (ATGCGTCGACACTCTGGGGATCCGACC). HA-tagged, LILR-PDGFR transmembrane constructs were then subcloned into the vector pMx puro which encoded the sequence of the cytoplasmic tail of the human CD3ζ chain (a gift from Dr Alex Barrow, St Louis USA) [4] using the primers NK1313 (ATGCTTAATTAATCCACCATGGAGACAGAC) and NK1314 (CTTCTCGAGCCAAAGCATGATGAGGATG).

Following transient transfection of PLAT-E retroviral packaging cells with the pMx HA-LILR-PDGFR-CD3ζ fusion constructs, recombinant retrovirus was used to transduce 2B4 T cell hybridoma cells that had previously been stably transfected with a NFAT-GFP reporter construct (a kind gift from Lewis Lanier, UCSF, San Francisco, California, USA) [5]. Hereafter these cells are referred to as 2B4 reporter cells. Following transduction, 2B4 reporter cells were sorted using a MoFlo cytometer (Beckman Coulter, Brea, CA, USA) for similar levels of expression of each LILR based on the level of expression of the LILR-fused N terminal HA epitope stained with an anti-HA-PE monoclonal antibody (Miltenyi Biotec, Bergisch Gladbach, Germany).

Preparation of cell lysates

Adherent cells were detached using non-enzymatic cell dissociation buffer (NECDB, Sigma-Aldrich, St. Louis, MO, USA) and then washed with PBS pH 7.4 before lysis. For whole cell lysates, each $1x10^7$ cells to be lysed were pelleted and resuspended in 100µl of 1% Nonidet P-40, 150 mM NaCl, 5 mM EDTA, 1mM $MgCl_2$, 50 mM Tris HCl pH 7.5, and 1x Proteoblock protease inhibitor cocktail (Fermentas, Vilnius, Lithuania) for 10 minutes on ice. Cell debris and nuclei were removed by centrifugation at 17000g at 4°C for 30 min; lysate supernatants were used immediately or snap frozen and stored at -80°C.

For lysates enriched for membrane proteins, each $1x10^8$ cells were resuspended in 1ml of cold PBS pH 7.4 containing 1mM $MgCl_2$. Cells were frozen and thawed twice and membrane proteins were pelleted by centrifugation for 30 min at 17000g at 4°C. Pellets were then resuspended and dissolved in the same lysis buffer used for whole cells.

shRNA silencing of cytokeratin 8 expression by MCF-7 cells

A lentiviral vector was used to stably transduce MCF-7 cells with shRNA construct designed to target human cytokeratin 8 mRNA. Transduction with an shRNA sequence with no target in the human genome was used as a negative control. shRNA oligomer pairs were annealed and ligated into AgeI/EcoRI cut pLKO-1-puro lentiviral vector (Addgene). HEK293T cells were transfected with the pLKO-1 constructs and the packaging plasmids pCMV-Δ8.9 and pMDG (a gift from Louise Boyle, CIMR, Cambridge). Supernatants containing virus were harvested after 4 days culture, 0.2µm-filtered and incubated with MCF-7 cells for 18hrs. Cells were subsequently placed under selection with 1.5µg/ml puromycin. Cells surviving selection and displaying low levels of cytokeratin 8 expression, as determined by the anti-human cytokeratin 8 monoclonal antibody 1E8, were sorted and cloned using a MoFlo cytometer (Beckman Coulter). Cytokeratin 8 expressing, puromycin resistant cells transduced with the control shRNA virus were maintained and used as bulk cultures.

Tissue culture conditions

All cell lines were cultured in RPMI 1640 supplemented with 10% FBS, penicillin-streptomycin (50 U/ml) and Amphotericin B (2.5 µg/ml) at 37°C in a 5% CO_2 in air atmosphere. Cell lines used were as follows: the EBV transformed B cell 721.221 (HLA-Class I deficient) both untransfected and stably transfected with HLA-G [1]; the EBV positive and negative Burkitt's lymphoma B cell lines Daudi (β_2 microglobulin deficient) and BJAB; the epithelial cell lines MCF-7 (breast glandular), T47D (breast ductal) and HCT-116 (colon); the human embryonic kidney cell line thought to be of neuronal origin HEK293T and the 2B4 cell line, a mouse T cell hybridoma stably transfected with a NFAT-GFP reporter construct (a kind gift from Lewis Lanier, UCSF, San Francisco, California, USA). Human breast epithelial cells of non-tumour origin were cultured as previously described [6]. All primary human material was derived from reduction mammoplasties carried out at Addenbrooke's NHS Trust, Cambridge, UK, under full informed consent and in accordance with Ethics Committee approval (08/H0308/178) as part of the Adult Breast Stem Cell Study.

SUPPLEMENTARY REFERENCES

1. Jones DC, Roghanian A, Brown DP, Chang C, Allen RL, Trowsdale J, Young NT. Alternative mRNA splicing creates transcripts encoding soluble proteins from most LILR genes. Eur J Immunol. 2009; 39:3195–3206.
2. Jones DC, Kosmoliaptsis V, Apps R, Lapaque N, Smith I, Kono A, Chang C, Boyle LH, Taylor CJ, Trowsdale J, Allen RL. HLA class I allelic sequence and conformation regulate leukocyte Ig-like receptor binding. J Immunol. 2011; 186:2990–2997.
3. Lopez-Alvarez MR, Jones DC, Jiang W, Traherne JA, Trowsdale J. Copy number and nucleotide variation of the LILR family of myelomonocytic cell activating and inhibitory receptors. Immunogenetics. 2014; 66:73–83.
4. Barrow AD, Raynal N, Andersen TL, Slatter DA, Bihan D, Pugh N, Cella M, Kim T, Rho J, Negishi-Koga T, Delaisse JM, Takayanagi H, Lorenzo J, Colonna M, Farndale RW, Choi Y, et al. OSCAR is a collagen receptor that costimulates osteoclastogenesis in DAP12-deficient humans and mice. J Clin Invest. 2011; 121:3505–3516.
5. Arase H, Mocarski ES, Campbell AE, Hill AB, Lanier LL. Direct recognition of cytomegalovirus by activating and inhibitory NK cell receptors. Science. 2002; 296:1323–1326.
6. Stingl J, Eirew P, Ricketson I, Shackleton M, Vaillant F, Choi D, Li HI, Eaves CJ. Purification and unique properties of mammary epithelial stem cells. Nature. 2006; 439:993–997.
7. Delport W, Poon AF, Frost SD, Kosakovsky Pond SL. Datamonkey 2010: a suite of phylogenetic analysis tools for evolutionary biology. Bioinformatics. 2010; 26:2455–2457.
8. Kosakovsky Pond SL, Frost SD. Not so different after all: a comparison of methods for detecting amino acid sites under selection. Mol Biol Evol. 2005; 22:1208–1222.
9. Pond SL, Frost SD, Grossman Z, Gravenor MB, Richman DD, Brown AJ. Adaptation to different human populations by HIV-1 revealed by codon-based analyses. PLoS Comput Biol. 2006; 2:e62.
10. Murrell B, Wertheim JO, Moola S, Weighill T, Scheffler K, Kosakovsky Pond SL. Detecting individual sites subject to episodic diversifying selection. PLoS Genet. 2012; 8:e1002764.
11. Murrell B, Moola S, Mabona A, Weighill T, Sheward D, Kosakovsky Pond SL, Scheffler K. FUBAR: a fast, unconstrained bayesian approximation for inferring selection. Mol Biol Evol. 2013; 30:1196–1205.

12. Saitou N, Nei M. The neighbor-joining method: a new method for reconstructing phylogenetic trees. Mol Biol Evol. 1987; 4:406–425.
13. Hasegawa M, Kishino H, Yano T. Dating of the human-ape splitting by a molecular clock of mitochondrial DNA. J Mol Evol. 1985; 22:160–174.
14. Sali A, Potterton L, Yuan F, van Vlijmen H, Karplus M. Evaluation of comparative protein modeling by MODELLER. Proteins. 1995; 23:318–326.
15. Wu S, Zhang Y. MUSTER: Improving protein sequence profile-profile alignments by using multiple sources of structure information. Proteins. 2008; 72:547–556.
16. Laskowski RA, MacArthur MW, Moss DS, Thornton JM. PROCHECK: a program to check the stereochemical quality of protein structures. J Appl Cryst. 1993; 26:283–291.
17. Zhou H, Zhou Y. Distance-scaled, finite ideal-gas reference state improves structure-derived potentials of mean force for structure selection and stability prediction. Protein science : a publication of the Protein Society. 2002; 11:2714–2726.
18. Benkert P, Biasini M, Schwede T. Toward the estimation of the absolute quality of individual protein structure models. Bioinformatics. 2011; 27:343–350.
19. Benkert P, Tosatto SC, Schomburg D. QMEAN: A comprehensive scoring function for model quality assessment. Proteins. 2008; 71:261–277.

Supplementary Table S1: Detection of significant evolutionary selection pressure acting upon individual codons within the sequence encoding the extracellular portion of *LILRB3* and *LILRA6*

	Codon	SLAC dN-dS	SLAC *p*-value	FEL dN-dS	FEL *p*-value	IFEL dN-dS	IFEL *p*-value	MEME dN-dS	MEME *p*-value	FUBAR dN-dS	FUBAR Post. Pr.	Summary				
												SLAC	FEL	IFEL	MEME	FUBAR
Positive Selection	36	7.222	0.077	187.057	0.013	214.034	0.018	30.61	0.021	10.259	0.998	A	ABD	AD	AD	ABD
	46	8.47	0.036	202.094	0.017	201.949	0.031	33.10	0.027	9.953	0.996	A	A	A	A	A
	97	4.914	0.199	127.367	0.126	199.734	0.072	299.66	<0.001	6.592	0.972			A	ABD	A
	164	5.887	0.126	158.443	0.088	103.633	0.190	26.08	0.113	8.53	0.980		A			AD
	182	10.904	0.042	270.696	0.033	313.122	0.032	74.35	0.041	12.892	0.992	AD	AD	AD	A	ABCD
	265	4.692	0.132	133.93	0.074	121.333	0.116	21.96	0.097	7.912	0.985		A			AD
	318	6.366	0.073	162.774	0.046	117.358	0.123	26.66	0.064	8.069	0.991	A	A			A
	327	6.175	0.157	158.277	0.063	241.983	0.034	6647.72	<0.001	8.651	0.990		A	A	A	A
	377	14.947	0.004	409.789	0.006	343.926	0.017	278.32	<0.001	15.626	0.996	AD	AD	AD	ACD	ABCD
	386	14.219	0.016	428.703	0.016	309.463	0.048	645.67	<0.001	15.687	0.991	A	A	A	ABCD	ABD
Negative Selection	70	-7.507	0.012	-169.353	0.005	-169.353	0.036	n/a	n/a	-8.98	0.999	A	AD	AD	n/a	AD
	98	-6.151	0.055	-209.643	0.009	-209.643	0.036	n/a	n/a	-9.872	0.992	AD	ABCD	ABD	n/a	AD
	217	-6.151	0.041	-305.265	0.003	-305.265	0.016	n/a	n/a	-11.103	0.995	A	A	A	n/a	A
	267	-14.125	0.035	-268.262	0.131	-322.083	0.100	n/a	n/a	-5.332	0.713	A		A	n/a	
	274	-27.68	<0.001	-520.286	<0.001	-520.286	<0.001	n/a	n/a	-15.926	1	A	ABD	A	n/a	A
	287	-3.076	0.203	-127.515	0.045	-127.515	0.101	n/a	n/a	-4.414	0.886		ABD	BD	n/a	
	326	-16.103	0.023	-972.993	0.001	-932.341	0.011	n/a	n/a	-10.911	0.975	A	AD	A	n/a	A
	392	-25.1	<0.001	-690.344	<0.001	-690.344	<0.001	n/a	n/a	-15.923	1	ABD	ABD	ABD	n/a	ABD

DataMonkey (http://www.datamonkey.org/) [7] was used to assess the influence of positive selection on individual codons within the extracellular Ig region of LILRB3 and LILRA6. Several tests on the DataMonkey server were used to assess selection pressure acting upon individual sites: Fixed effects likelihood (FEL) [8]; internal fixed effects likelihood (IFEL) [9]; single likelihood ancestor counting (SLAC) [8]; mixed effects model of evolution (MEME) [10]; and fast unconstrained Bayesian approximation (FUBAR) [11]. Neighbour joining (NJ) phylogenetic trees [12] and the HKY85 model of evolution [13] were employed in all tests.

Analysis was initially performed on an alignment consisting of the cDNA sequences of all LILRB3 and LILRA6 alleles described in [3], together with previously reported sequences (Supplementary Table S2) deposited in Genbank (http://www.ncbi.nlm.nih.gov/genbank/) (total number of sequences n=50; alignment A). Genbank sequences that contained unique substitutions were excluded from the alignment to avoid confusion from possible sequence errors. The p values and normalised dN-dS values are given in the left side of the table. A significance threshold of $p<0.05$ was used in the case of MEME, while a threshold of $p<0.1$ was used for the more conservative SLAC, FEL and IFEL tests. Results from FUBAR were considered significant when a posterior probability (Post. Pr.) of >0.95 was achieved.

Three further sequence alignments were analysed, consisting of: B) LILRB3 alleles sequenced in [3] (n=14); C) LILRA6 alleles [3] (n=11); and D) the combined alleles of LILRB3 and LILRA6 [3] (n=25). The summaries of these analyses are provided in the right side of the table, where a statistically significant result is denoted by a letter (A-D) that corresponds to the alignment in which it was detected. Only Codons that were discovered to be under significant selection by two or more tests are shown.

Supplementary Table S2: Previously reported *LILRB3* alleles included in tests for evolutionary selection pressure

AF344652	AF009636	AF344442	AF009641	AF009642
AF344428	AF009634	AF344437	AK290917	NM_006864
AF009632	AF344651	AF009635	AF344435	AF031555
AF344438	AF031554	AF344440	AF344430	AF344429
AF344441	AF344443	AF344434	AF025533	AF009638

Accession numbers are provided. Sequences published by Lopez-Alvarez *et al*. [3] are not listed.

Supplementary Table S3: Results of Model Evaluation: D1-D2

Method/ Server	Template	Ramachandran plot statistics		G-Factor scores			Dfire energy (kJ/mol)	Overall QMEAN6 Z score
		Residues in most favoured positions	Residues in disallowed positions	Average Dihedral Angles	Average Main-chain Covalent Forces	Overall Average G Factors		
MODELLER	1g0x:A (LILRB1, D1-D2)	**95.5%**	0.6%	-0.09	-0.31	**-0.17**	**-240.64**	**-1.63**
MODELLER	3p2t:A (LILRB4, D1&D4)	**92.9%**	0.0%	-0.12	-0.42	**-0.23**	**-233.33**	**-1.58**
MODELLER	3d2u:D (LILRB1, D1-D2)	**92.3%**	0.6%	-0.07	-0.27	**-0.14**	**-232.34**	**-1.14**
MODELLER	2d3v:A (LILRA5, D1-D2)	**89.7%**	2.6%	-0.21	-0.60	**-0.35**	**-222.97**	**-0.87**
MODELLER	2dyp:D (LILRB2, D1-D2)	**87.9%**	0.0%	-0.14	-0.26	**-0.18**	**-233.33**	**-1.09**
MODELLER	1p6f:A (NKp46)	**86.0%**	0.6%	-0.26	-0.36	**-0.29**	**-228.43**	**-1.69**
RaptorX	3p2t:A, 2d3v:A	**95.5%**	0.6%	-0.16	0.62	**0.16**	**-249.57**	**-0.92**

Single-template homology models of Ig domains D1 and D2 of LILRB3*12 were produced based on the related structures 1g0x:A and 3d2u:D (LILRB1 D1-D2), 2dyp:D (LILRB2 D1-D2), 3p2t:A (LILRB4 D1 and D4), 2d3v:A (LILRA5 D1-D2) and 1p6f:A (NKp46) using the MODELLER program [14] via the Max-Planck Bioinformatics Toolkit (http://toolkit.tuebingen.mpg.de/modeller). This approach was used to produce homology models of LILRB3 D3-D4 based on the LILR templates 3p2t:A (LILRB4 D1 and D4), 4ll9:A (LILRB1 D3-D4) and 4lla:A (LILRB2 D3-D4) and the related KIR structures 1b6u:A (KIR2DL3) and 1efx:D (KIR2DL2). Structurally related non-LILR templates were identified and aligned using MUSTER [15].

Supplementary Table S4: Results of Model Evaluation: D3-D4

Method/ Server	Template	Ramachandran plot statistics		G-Factors scores			Dfire energy (kJ/mol)	Overall QMEAN6 Z score
		Residues in most favoured positions	Residues in disallowed positions	Average Dihedral Angles	Average main-chain Covalent Forces	Overall Average G Factors		
MODELLER	4lla:A (LILRB2 D3-D4)	**95.0%**	0.6%	-0.08	-0.31	**-0.16**	**-232.79**	**-0.44**
MODELLER	4ll9:A (LILRB1 D3-D4)	**90.5%**	0.6%	-0.16	-0.29	**-0.20**	**-223.06**	**-0.93**
MODELLER	3p2t:A (LILRB4, D1&D4)	**91.4%**	0.0%	-0.13	-0.33	**-0.20**	**-219.83**	**-1.19**
MODELLER	1efx:D (KIR2DL2)	**85.8%**	0.6%	-0.16	-0.34	**-0.22**	**-221.13**	**-0.57**
MODELLER	1b6u:A (KIR2DL3)	**85.8%**	1.2%	-0.21	-0.43	**-0.28**	**-221.69**	**-1.18**
RaptorX	4lla:A (LILRB2 D3-D4)	**94.4%**	0.6%	-0.12	-0.31	**-0.18**	**-233.7**	**-0.50**

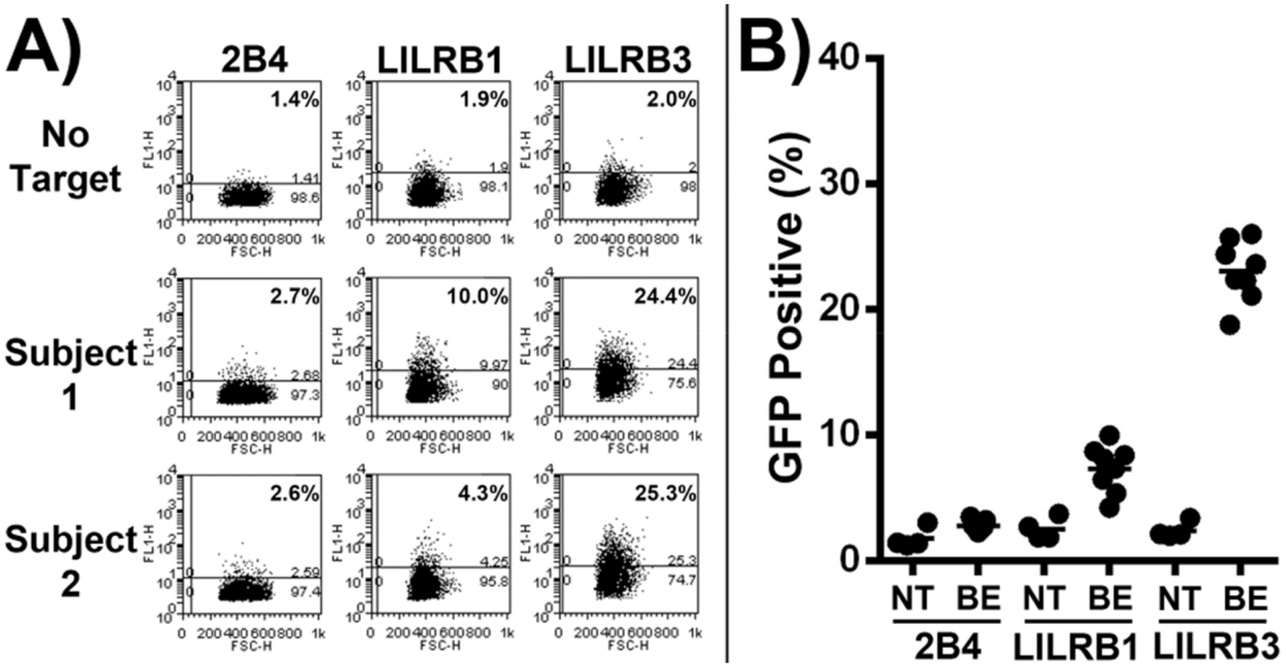

Supplementary Figure S1: Activation of LILRB3 2B4 reporter cells by human non-tumour breast epithelial cells. A. Flow cytometry dot plots of GFP expression by parental 2B4 reporter cells, and reporter cells expressing either LILRB1 or LILRB3*12, following incubation with either normal human breast epithelial cells cultured from two subjects or without target cells. **B.** GFP expression by reporter cells incubated with normal human breast epithelial cells (BE) cultured from four subjects or without target cells (NT). Breast epithelial cells from each subject were tested in duplicate.

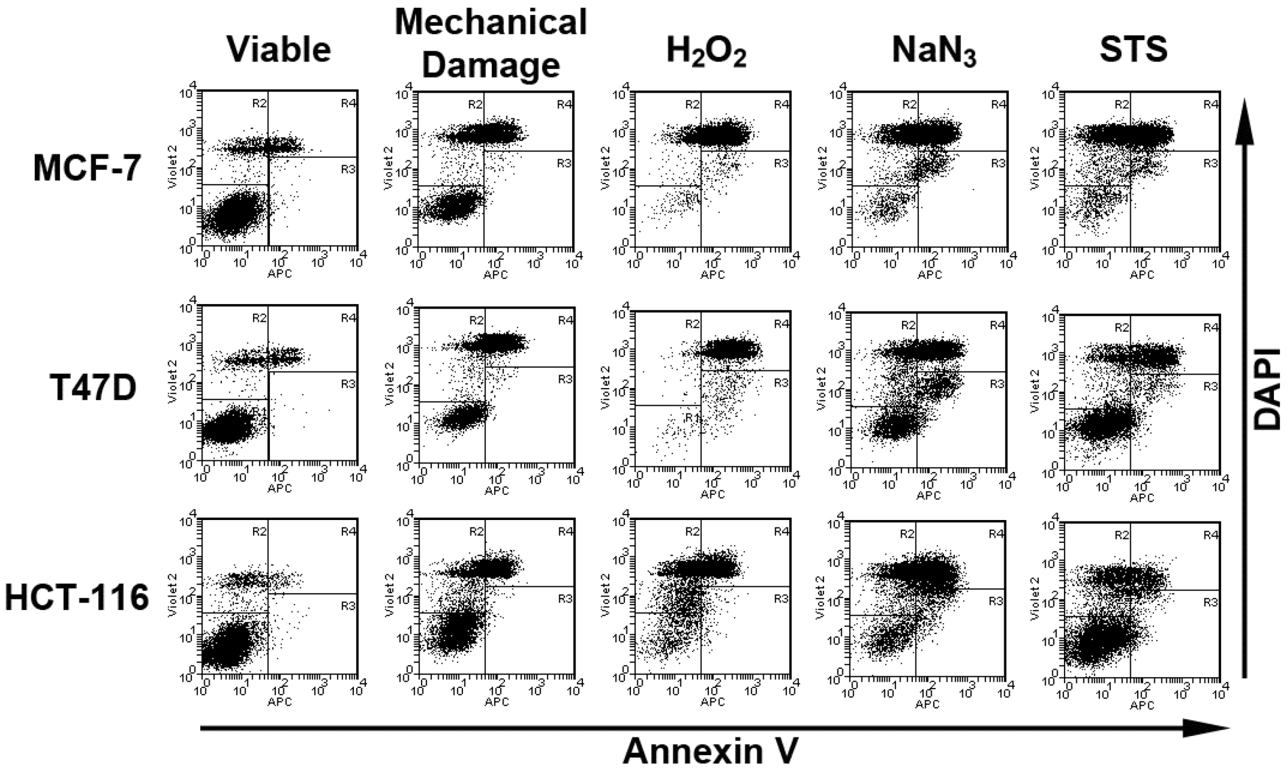

Supplementary Figure S2: Assessment of cell death by Annexin V and DAPI staining. FACS plots are divided into four regions: bottom left contains viable cells, top right are either late apoptotic or necrotic, while bottom right are early apoptotic.

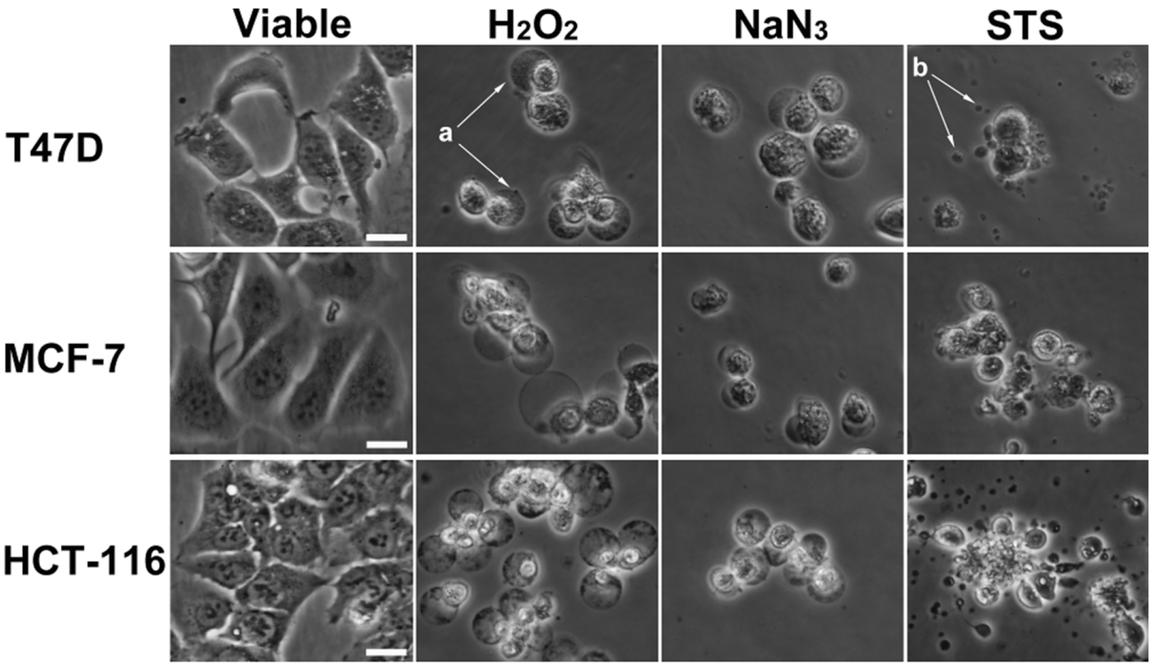

Supplementary Figure S3: Assessment of the Morphology of tumour cell lines following treatment with necrotic and apoptotic agents. T47D, MCF-7 and HCT-116 cells were treated with either H_2O_2, NaN_3 or STS. Using a light microscope, cells were assessed for the characteristics of necrosis (cellular swelling [arrowed a]) and apoptosis (cell shrinkage and formation of apoptotic bodies [b]). Bar represents 100μm.

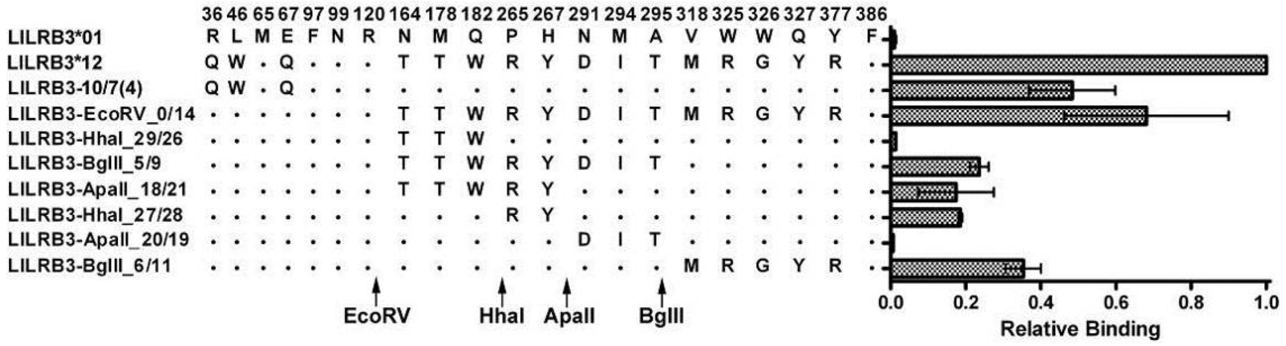

Supplementary Figure S4: Variation in LILRB3 affects binding to mechanically damaged MCF-7 cells. Restriction fragments were used to produce a panel of chimeric LILRB3-Fc molecules from the sequence of two alleles, LILRB3*01 and LILRB3*12. Dots (.) denote consensus with the LILRB3*01 sequence.

Chimeric LILRB3-Fc fusion proteins were used to stain epithelial MCF-7 cells and the results from each chimeric LILRB3-Fc are shown to the right of the sequence. Mean relative binding values and standard deviations from 8 independent experiments are shown. Relative binding values were calculated by dividing the mean fluorescence intensity (MFI) of the high binding LILRB3*12 allele by each of the other Fc molecules tested. No binding to the negative control cell line HEK293T was observed (data not shown).

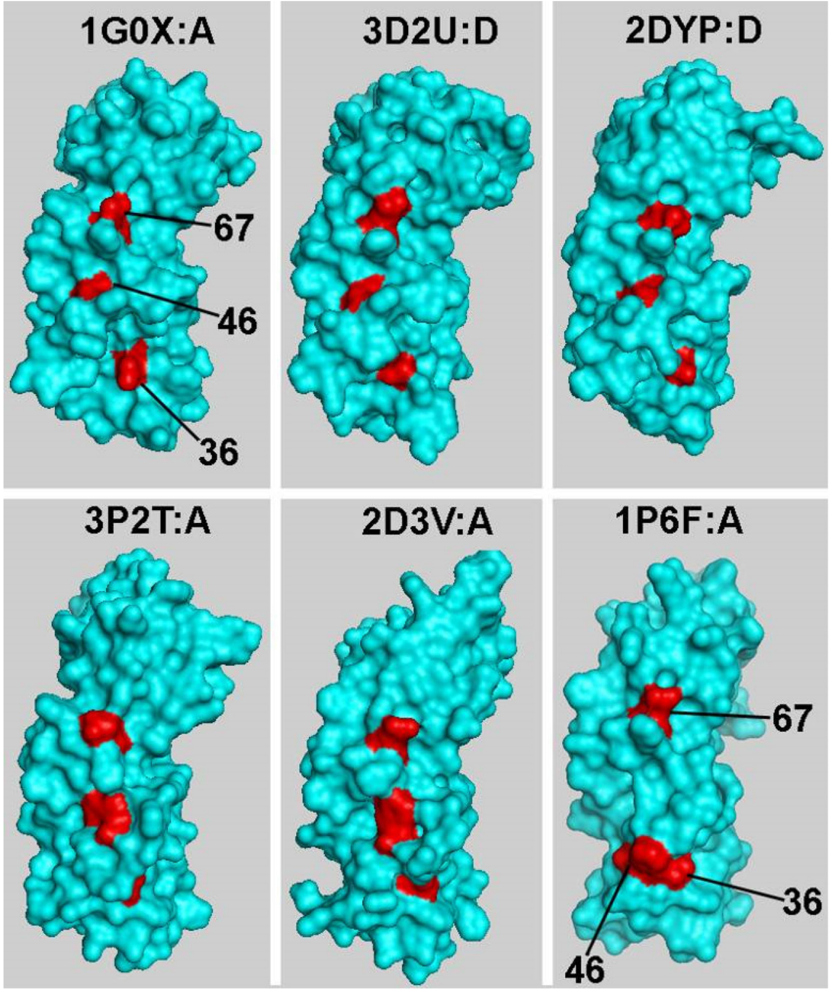

Supplementary Figure S5: Homology modelling of D1-D2 LILRB3/A6 using single template structures. All models were predicted using MODELLER based on the following LILR crystal structures: 1g0x:A and 3d2u:D (LILRB1, D1-D2), 2dyp:D (LILRB2, D1-D2), 3p2t:A (LILRB4, D1&D4), 2d3v:A (LILRA5, D1-D2) and the more distantly related structure 1p6f:A (NKp46). The polymorphic residues 46 and 67 influenced binding of necrotic glandular epithelial cells and are shown in red. Residue 36 is also highlighted; polymorphism at this site displays strong linkage disequilibrium with residue 67. Information regarding the quality evaluation of each model is provided in Supplementary Table S3.

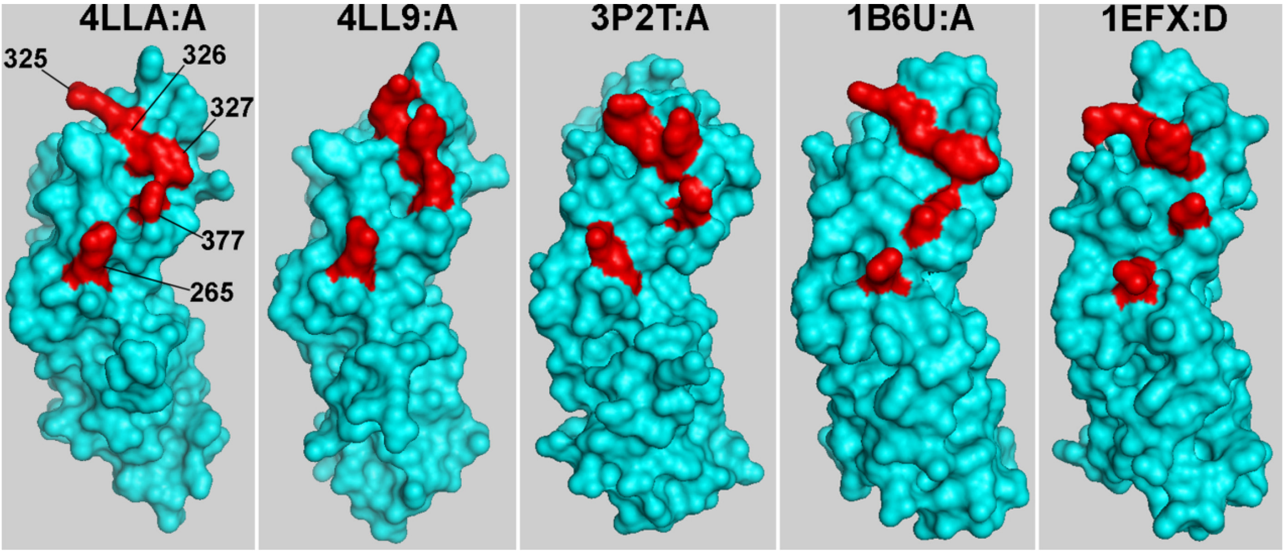

Supplementary Figure S6: Predicted protein structures of D3-D4 of LILRB3/A6 based on single temples. Models were generated by MODELLER using the LILR templates 4lla:A (LILRB2 D3-D4), 4ll9:A (LILRB1 D3-D4) and 3p2t:A (LILRB4, D1&D4), in addition to the related KIR structures 1b6u:A (KIR2DL3) and 1efx:D (KIR2DL2). Sites identified as interacting with necrotic glandular epithelial cells (namely residue 265 within D3, and 325-7 and 377 within D4) are highlighted in red. Information regarding the quality evaluation of each model is provided in Supplementary Table S4.

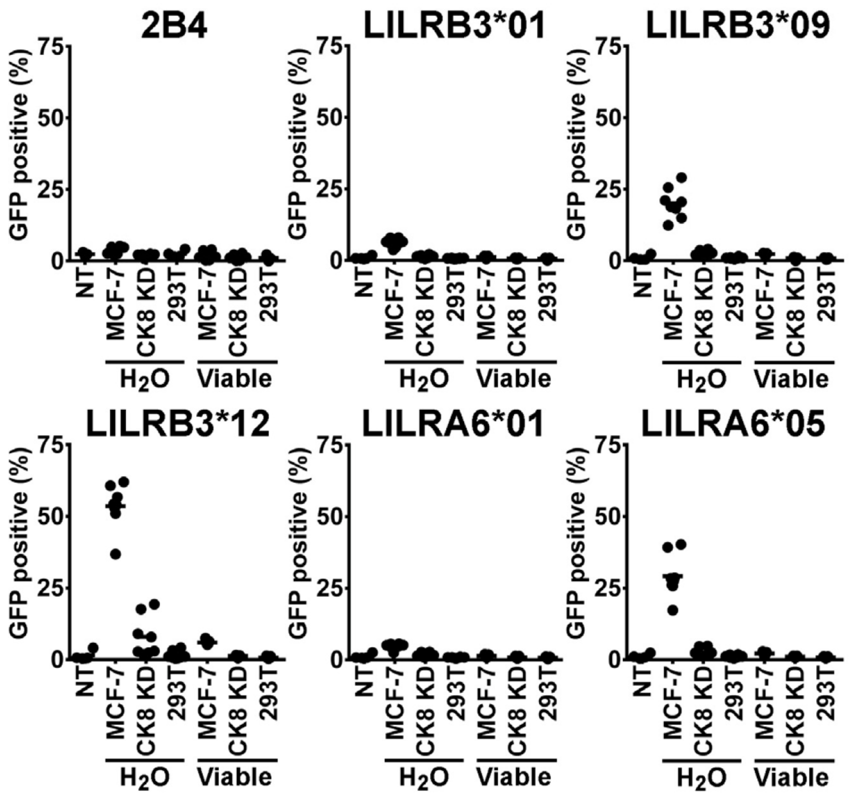

Supplementary Figure S7: Silencing of cytokeratin 8 expression abrogates cellular recognition of osmotically lysed breast cancer cells by LILRB3 and -A6 allelic variants. 2B4 reporter cells and 2B4 cells transfected with naturally occurring LILRB3 and LILRA6 variants were incubated with either viable target cells, target cells previously treated with H_2O to induce osmotic lysis, or without target cells (NT). The target cell lines assessed were MCF-7 cytokeratin 8 knock down cells (CK8 KD), MCF-7 cells transduced with a non gene-specific shRNA construct and HEK293T. The results shown are from 4 replicate experiments, where each assessment was performed in duplicate. Bars indicate mean values.
